# Supplementary material for: Validation of IL-7R as an Immunological Biomarker for Human Pancreatic Ductal Adenocarcinoma
Source: Cancers (Basel). 2022 Feb 8;14(3):853. doi: 10.3390/cancers14030853 (PMC8834093; doi:10.3390/cancers14030853)
Supplement: Supplementary file 1 [file cancers-14-00853-s001.zip › cancers-1550971-supplementary.pdf]

**Supplementary Table S1. Primers used in the qRT-PCR.**

| Gene | Primer | Sequence |
| --- | --- | --- |
| | Forward | 5′-CTG GAG AAA GTG GCT ATG CTC-3′ |
| *hIL-7RA* | Reverse | 5′-ACA TCT GGG TCC TCA AAA GC-3′ |
| | Probe-FAM | 5′-CAG TTG GAA GTG AAT GGA TCG CAG C-3′ |
| | Forward | 5′-CCA AGG TCA TCC ATG ACA ACT-3′ |
| *hGAPDH* | Reverse | 5′-ATC ACG CCA CAG TTT CCC-3′ |
| | Probe-Joe | 5′ATC ACT GCC ACC CAG AAG ACT GTG-3′ |
